# Coronavirus genome packaging and nucleocapsid assembly

**DOI:** 10.1128/jvi.01330-25

**Published:** 2026-01-08

**Authors:** Paul S. Masters

**Affiliations:** 1 Division of Infectious Diseases, Wadsworth Center, New York State Department of Health, Slingerlands, New York, USA; 2 Department of Biomedical Sciences, University at Albany 1084, Albany, New York, USA; Indiana University Bloomington, Bloomington, Indiana, USA

**Keywords:** coronavirus, genomic RNA packaging, packaging signal, nucleocapsid assembly, protein phosphorylation

## Abstract

Coronaviruses are a family of positive-strand RNA viruses that exhibit highly selective packaging of their genomic RNA (gRNA) into assembled virions, despite the presence of a large excess of subgenomic viral RNA species and host RNA in infected cells. While this high selectivity is critical to evading host innate immune responses, surprisingly, it is not essential for virion assembly. This review focuses on four main topics: (i) coronavirus genome packaging signals (PSs)—how they are found and the function they serve; (ii) the viral components that recognize the PS in order to bring about selective gRNA packaging; (iii) coronavirus nucleocapsid structure and assembly; and (iv) the relationship between nucleocapsid protein phosphorylation and nucleocapsid assembly versus RNA synthesis. Current understanding of these areas has benefited immensely from advances made by recent studies, most of which were performed in response to the emergence of the coronavirus responsible for the COVID-19 pandemic. Throughout this review, emphasis is placed on the counterintuitive distinction between coronavirus selective gRNA packaging and nucleocapsid assembly.

## CORONAVIRUSES

Most RNA viruses selectively package their genomes into virions and exclude other viral RNA species generated during the course of infection, as well as cellular RNAs. This specificity is accomplished through various strategies, each dictated by the molecular details of individual viral replication pathways. Coronaviruses are a family of positive-strand RNA viruses in which there are four genera*—Alpha-*, *Beta-*, *Gamma-*, and *Deltacoronaviruses*—that infect mammalian or avian hosts (1). Among these, the *Betacoronaviruses* have been subjected to intense scrutiny because they contain severe acute respiratory syndrome coronavirus 2 (SARS-CoV-2), the causative agent of COVID-19. The *Betacoronaviruses* also include two other dangerous human pathogens, SARS-CoV and Middle East respiratory syndrome coronavirus (MERS-CoV), as well as two endemic human coronaviruses (HCoVs), HCoV-OC43 and HCoV-HKU1.

**Fig 1:**
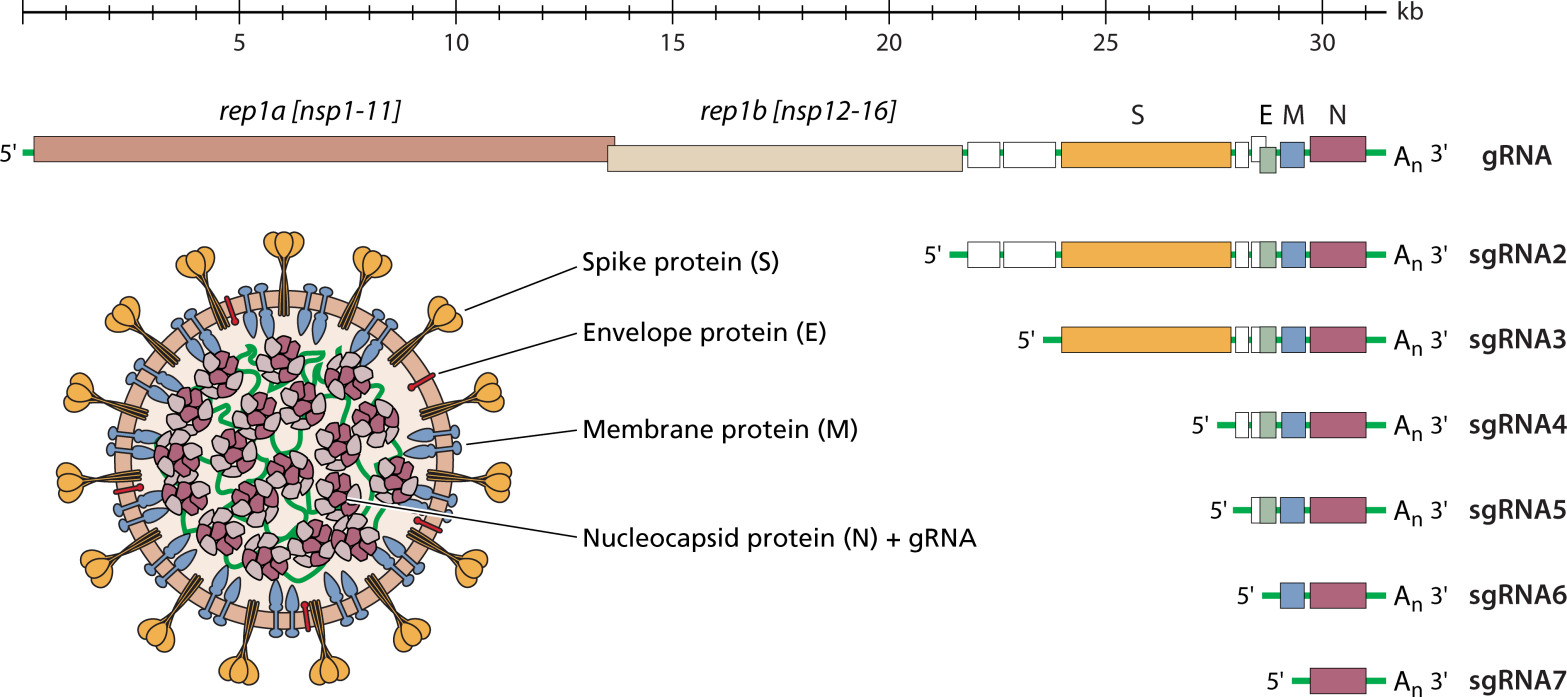
Coronavirus RNA and virion composition. Top, the coronavirus genome (gRNA), as exemplified by mouse hepatitis virus. The 5′ end consists of the rep1a and rep1b genes encoding the viral replicase-transcriptase; the 3′ end contains structural protein genes (S, E, M, and N), as well as accessory genes (unlabeled). Beneath the gRNA is the 3′-nested set of subgenomic mRNAs (sgRNAs) synthesized during infection. Left, a cartoon of the coronavirus virion, showing the canonical set of structural proteins: spike (S), membrane (M), envelope (E), and nucleocapsid (N) proteins.

Coronavirus virions are roughly spherical particles, ~85–100 nm in diameter, containing a minimal set of four structural proteins (Fig. 1). Three of these are integrated into a host-derived membrane envelope: spike (S) protein, which binds to receptors and mediates viral entry into the host cell cytoplasm (2); membrane protein (M), which promotes membrane curvature and plays a central role in virion assembly; and envelope protein (E), a small ion channel that assists M in envelope formation. In the interior of the virion, multiple copies of the nucleocapsid protein (N) wrap the viral genome into tightly packed ribonucleoprotein clusters resembling beads on a string.

The coronavirus genome (gRNA) is a single-stranded, positive-sense RNA molecule that is polycistronic and unusually long (~30 kb for *Betacoronaviruses*) but otherwise resembles a standard eukaryotic mRNA, with a 5′ cap and a 3′ polyadenylate tail. Once it is delivered to the cytoplasm, gRNA serves as the first mRNA of infection. Translation of the rep1a and rep1b genes in the 5′ two-thirds of the genome (Fig. 1), via a ribosomal frameshifting mechanism, results in two huge, partially overlapping polyproteins (pp1a and pp1ab). These undergo cotranslational autoproteolytic processing into 16 nonstructural proteins (nsps) that constitute the viral replicase-transcriptase complex (RTC), including components that remodel intracellular membrane structures to house this complex (1). Coronavirus RNA synthesis is an elaborate process, the hallmark of which is the production of a 3′-nested set of subgenomic RNAs (sgRNAs), which act as the mRNAs for the downstream-encoded genes. Each sgRNA contains a short (70–100-nt) 5′ leader sequence, which is identical to the 5′ end of the genome, joined to a 3′ body sequence, which is identical to the 3′ end of the genome (Fig. 1). The fusion event that creates each sgRNA is brought about during negative-strand RNA synthesis through a discontinuous step that occurs at transcription-regulating sequences, short motifs that appear at the end of the leader and at the start of the body segment (3, 4). Following the translation of a sufficient pool of structural proteins, assembled nucleocapsids merge with the membrane-bound proteins, budding into the endoplasmic reticulum-Golgi intermediate compartment to form progeny virions that are exported via exocytic vesicles or lysosomal trafficking (5–7).

Key to the subject of this review is that, while infection generates abundant quantities of various sgRNAs, only full-length positive-sense gRNA ultimately becomes packaged into virions. Highly selective gRNA packaging appears to be a universal property of coronaviruses, although this has not been exhaustively investigated, because some coronaviruses grow poorly in tissue culture or else are very hazardous to manipulate in quantities required for stringent purification. It is thus especially noteworthy that selective gRNA packaging has recently been rigorously demonstrated for SARS-CoV-2 (8).

## CORONAVIRUS GENOME PSs

A PS, broadly defined, is an RNA sequence and structure that specifies the selective incorporation of gRNA into assembled virions. Among coronaviruses, the most thoroughly characterized PS is that found in the *Embecovirus* subgenus, one of the five subgenera of the *Betacoronaviruses*. The *Embecoviruses*, typified by mouse hepatitis virus (MHV), also include bovine coronavirus (BCoV), HCoV-OC43, and HCoV-HKU1. SARS-CoV-2 and SARS-CoV fall into a separate *Betacoronavirus* subgenus, the *Sarbecoviruses*.

### MHV and other *Embecoviruses*

**Fig 2:**
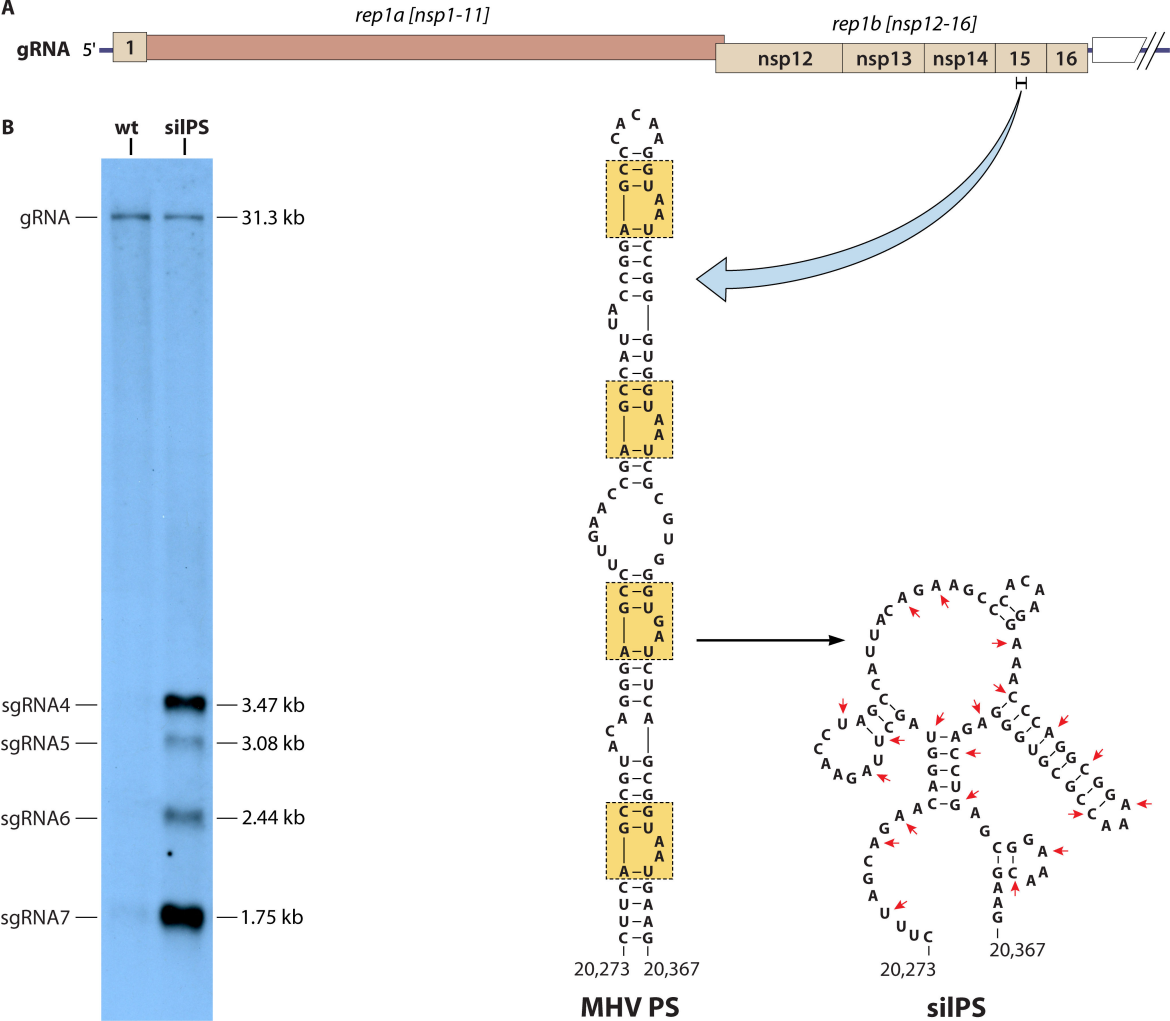
Genomic locus, structure, and mutagenesis of the MHV PS. (**A**) At the top, the portion of the MHV genome that is unique to gRNA is shown; the bracket indicates the central segment of the nsp15 gene containing the PS. Below this is the structure of the wild-type (wt) MHV PS (14), with the four repeat units boxed. To the right is the lowest free-energy mfold structure of the mutant silPS (15); red arrowheads denote the 20 coding-silent mutations that were made to disrupt the PS while preserving the nsp15 protein sequence. Genomic nucleotide coordinates are those of MHV-A59 (GenBank accession number AY700211). (**B**) Northern blot of RNA isolated from purified virions of wt MHV and the silPS mutant. The positions of gRNA and the more prominent sgRNAs are indicated on the left; the sizes of RNA species are given on the right. Viral RNA was detected with a probe corresponding to the N gene, which is common to gRNA and all sgRNAs.

The MHV PS was discovered through the manipulation of defective-interfering (DI) RNAs. Coronavirus DI RNAs (sometimes called defective RNAs or minigenomes) are extensively deleted derivatives of gRNA that retain *cis*-acting elements necessary for RNA synthesis. They replicate by exploiting the RTC provided by the helper virus, in many cases at the expense of the helper virus. A subset of MHV DI RNAs was found to be efficiently packaged by helper virus, and their dissection, guided by comparison with unpackaged DI RNAs, led to the mapping of the MHV PS to a portion of rep1b encoding part of nsp15 (9, 10) (Fig. 2A). A finer analysis used deletion and point mutants to confine the PS locus to a 190-nt segment of this region (11). Although this segment contained a 69-nt bulged stem-loop (SL) sufficient to function as a minimal PS, later studies found that larger regions encompassing this substructure were required for optimal efficiency (12, 13). Consistent with its role, the PS falls at a position roughly 20 kb from the 5′ end of the genome that is unique to gRNA and is not found in any sgRNA.

A structural model, developed using mfold (16) with manual adjustment, showed the MHV PS as a 95-nt SL with four repeat units, each presenting an AA (or GA) bulge on its 3′ side (14) (Fig. 2A). An internal loop between the second and third repeats divides the PS into quasi-symmetric upper and lower halves. This structure was supported by *in vitro* enzymatic and chemical probing (14). It was also found to be strongly conserved among the *Embecoviruses*, including species that were subsequently discovered (17). In all, the apical loop is invariant, while stem segments are preserved by covariance of base pairs; in a few cases, there are less than four repeat units, and the central loop is truncated.

The role of the PS in the intact viral genome was initially examined by constructing a mutant, designated silPS, in which 20 coding-silent mutations were generated to totally disrupt the structure of the PS while leaving the amino acid sequence of nsp15 unchanged (15) (Fig. 2A). Since prior DI RNA studies had created the notion that the PS was required for the incorporation of gRNA into virions, it was expected that the silPS mutant would be severely or lethally impaired. Surprisingly, the opposite result was observed. The silPS mutant was indistinguishable from the wild type in plaque size and growth kinetics in tissue culture, and highly purified virions of each had the same complement of structural proteins and the same particle-to-PFU ratio. A small fitness advantage of the wild type over the silPS mutant could only be demonstrated by competition through serial passaging. The striking difference between the two viruses was that silPS virions packaged abundant amounts of sgRNAs in addition to gRNA, whereas wild-type virions almost exclusively packaged gRNA (Fig. 2B). A similar phenotype—loss of gRNA packaging selectivity with little to no effect on viral growth—was obtained with an MHV mutant in which sequences encoding an epitope tag and its complement replaced most of the PS (18). Since the MHV PS codes for an unstructured loop on the surface of the nsp15 molecule (19, 20), it was also possible to delete it entirely, which produced a virus with the same phenotype as the silPS mutant (15). Moreover, when a transposed copy of the PS was inserted into the deletion mutant at a site immediately downstream of rep1b, the wild-type packaging phenotype was restored. This showed that the PS does not have to be situated in nsp15 and does not have to be translated in order to be functional.

Given that the growth of PS mutants in tissue culture was undisturbed, it was puzzling why the PS had evolved and what advantage was provided by the selective packaging of gRNA. This question was resolved by a study in which the 20 silPS point mutations were constructed in a highly neurovirulent strain of MHV (MHV-JHM), which resulted in dramatic attenuation of that virus (21). Mice infected with silPS MHV-JHM had diminished weight loss and a greatly increased rate of survival compared to wild-type-infected mice. Additionally, this attenuation was shown to be dependent on type-I interferon signaling, suggesting that sgRNAs conveyed into cells by the silPS mutant display a pathogen-associated molecular pattern that is absent or hidden in gRNA.

Two important principles thus emerged from studies that employed engineered viral mutants to investigate the role of the PS. First, selective gRNA packaging during coronavirus replication does not drive the assembly of virus particles. All PS mutants examined thus far were fully assembly-competent, despite failing to discriminate between sgRNA and gRNA. Second, while selective gRNA packaging is not essential in tissue culture, it is crucial *in vivo* to enable the virus to counter host innate immunity mechanisms.

### SARS-CoV and SARS-CoV-2

**Fig 3:**
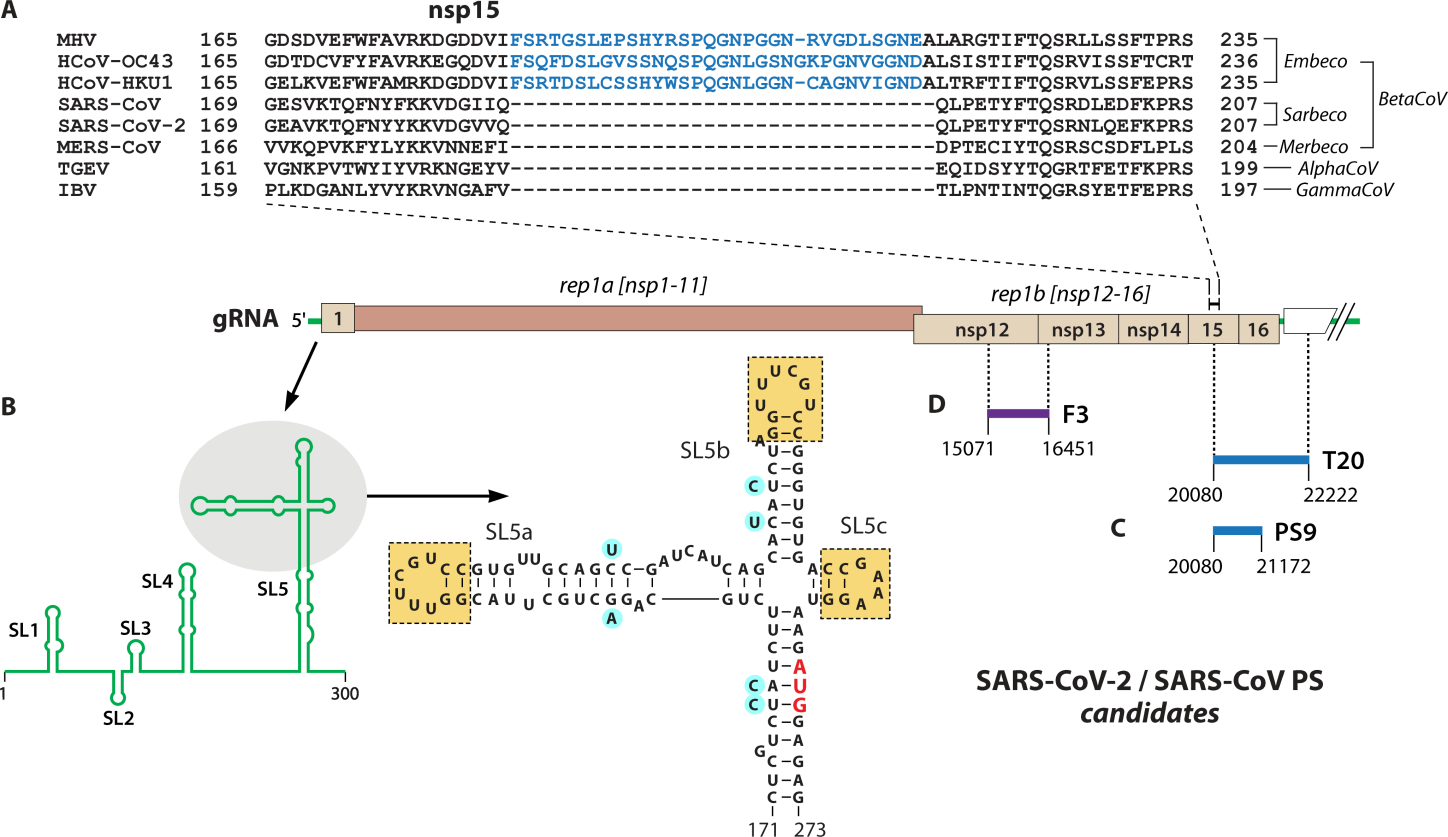
PS candidates for SARS-CoV and SARS-CoV-2. (**A**) Amino acid sequence alignment of the central portion of nsp15 of representative coronaviruses from different genera and subgenera. Highlighted in blue is the surface loop encoded by the *Embecovirus* PS, which is absent from all other members of the family. GenBank accession numbers for the sequences are: MHV, AY700211; HCoV-OC43, AY903460; HCoV-HKU1, AY597011; SARS-CoV, AY278741; SARS-CoV-2, MN908947; MERS-CoV, JX869059; transmissible gastroenteritis virus (TGEV), AJ271965; and infectious bronchitis virus (IBV), AJ311317. (**B**) The portion of the SARS-CoV-2 genome that is unique to gRNA. Below and to the left is a schematic of the structure of the 5′ end of gRNA, depicting the SLs SL1–SL5. To the right is an expansion of the trifurcated part of SL5 with boxes indicating the repeat units in SL5a and SL5b, as well as the third substructure, SL5c (17). Variant nucleotides in SARS-CoV are highlighted in blue; the start codon for rep1a is shown in red. (**C**) Genomic locations of SARS-CoV-2 PS candidate T20 and its derivative, PS9, which were identified by their highly active promotion of inclusion of a luciferase reporter into VLPs (25). (**D**) Genomic location of SARS-CoV-2 PS candidate F3, which was found through dissection of an efficiently packaged DI RNA (8). (**B–D**) All nucleotide coordinates are those of SARS-CoV-2.

It is clear that a homolog of the *Embecovirus* PS, and the surface loop it encodes (Fig. 3A), is not contained in *Sarbecoviruses* or other coronaviruses (17, 22). Although early reports showed an mfold-generated structure in the SARS-CoV nsp15 gene, PS580, that was said to resemble the MHV PS (23, 24), the basis for that assertion is obscure. Currently, there are three principal candidates for the genomic location of the *Sarbecovirus* PS. Due to the very high sequence identity between SARS-CoV and SARS-CoV-2 in all known *cis*-acting elements of gRNA, it is reasonable to assume that both have the same PS.

The first PS candidate was put forward based on considerations of RNA structure. Running from the 5′ untranslated region (5′ UTR) into the start of rep1a, coronavirus genomes all contain a series of SLs, some of which are critical for RNA synthesis. A comparative bioinformatics analysis of this region in SARS-CoV gRNA found that one of these, SL5, straddles the boundary of the 5′ UTR and nsp1 (22). The stem of SL5 continues to a four-way junction crowned by three SLs exhibiting a 5′-UUU(C/U)GU-3′ motif in two of the loops (Fig. 3B). This SL5 configuration, with two or three 5′-UUYYGU-3′ repeats, is conserved in most other *Beta-* and *Alphacoronaviruses*, and it has been confirmed by secondary structure probing for many of them, most recently SARS-CoV-2 (26–29). Noting that repeated sequence or structural motifs often can be PS attributes (14, 30, 31), it was argued that the SL5 extension constitutes a plausible PS. This branched structure is located in a region, well downstream of the 5′ leader, that is found only in gRNA and not in sgRNAs. Intriguingly, MHV and other *Embecoviruses*, which use the entirely distant PS described above, have a truncated form of SL5 lacking the four-way junction. As yet, this proposed PS has not been systematically tested, but there is some evidence that SL5 can induce the incorporation of an engineered DI RNA into SARS-CoV-2 virions (32). Three-dimensional models of the SARS-CoV-2 SL5 have been determined by cryo-EM, NMR, and crystallography (33–36). These reveal a T-shaped structure with the two 5′-UUU(C/U)GU-3′ loops at opposite ends of a coaxial stack of stems SL5a and SL5b (the crossbar of the T-shape). This structure, with some minor differences, is conserved in multiple coronaviruses. Of interest, the SL5 structure sequesters the start codon of rep1a, suggesting it could have a role analogous to the 5′ cap sequestration that identifies the particular HIV genomic transcript conformer chosen for packaging rather than translation (37).

A second SARS-CoV-2 PS candidate arose from the establishment of a virus-like particle (VLP) system devised to readily test the significance of mutations in evolved variants of this virus (25). Coronavirus VLPs are secreted particles generated by the eukaryotic expression of coronavirus structural proteins. They have the same size and morphology as virions and offer the advantage of being noninfectious and easier to manipulate. Early studies demonstrated that expression of merely the M and E proteins was sufficient for the formation of coronavirus VLPs (38), but subsequent work showed that addition of the N protein greatly increased the efficiency of VLP production (39–41). Inclusion of the S protein, although not required, allowed single-step passage of VLPs, and it was found that expressed RNA containing a PS could also be transferred to recipient cells (42). For the SARS-CoV-2 VLP system, 2-kb segments of the viral RNA were individually attached to an expressed luciferase mRNA reporter, and luciferase activity produced in recipient cells was measured. Importantly, the trialed RNA segments overlapped each other and comprehensively spanned the entire SARS-CoV-2 genome. Among these, many regions of rep1a and rep1b produced some level of luciferase activity, representing RNA incorporation into VLPs. However, a single segment, designated T20, derived from the downstream end of rep1b, stood out as having markedly higher activity than the others (Fig. 3C). Further dissection of T20 yielded a fragment with still higher activity, PS9, which mapped to a 1.1-kb region encompassing parts of the nsp15 and nsp16 coding regions. Although it partially overlaps PS9, the previous putative PS580 (24) showed little activity in the VLP system. It is also noteworthy that the 5′ genomic RNA segment containing SL5 had minimal activity. This study thus concluded that PS9 constitutes a sufficient element for RNA packaging into VLPs, but it could not yet be established whether it was required for the packaging of gRNA into virions.

A third SARS-CoV-2 PS candidate originated from the isolation of an efficiently packaged DI RNA obtained by extended serial passaging of SARS-CoV in tissue culture (8). This DI RNA turned out to be composed of short 5′ and 3′ end segments fused to an internal segment that ran from the coding region of nsp7 through nsp13. A set of smaller SARS-CoV-2 counterparts of this DI RNA was constructed, each containing the same 5′ and 3′ genomic end segments connected to one of seven partially overlapping genomic fragments covering nsp7 through nsp16. When tested with SARS-CoV-2 as helper virus, all of these were replication-competent, but only one, with an insert designated F3, was packaged into SARS-CoV-2 virions. The F3 insert corresponded to a 1.4-kb genomic segment crossing from nsp12 into nsp13 (Fig. 3D). The packaging capability of this segment was abolished by the introduction of multiple coding-silent mutations across its entirety or separately into each of three subregions. This may indicate that the PS comprises structures throughout the whole length of F3 or else that more fine-scale mutagenesis would be required to parse its makeup. Six SLs were identified in F3 that were similar between SARS-CoV-2 and SARS-CoV, but it was pointed out that none contain repeated structural motifs, as in the *Embecovirus* PS. It is interesting that a genomic region spanning the F3 segment is found in naturally occurring SARS-CoV-2 DI RNAs that are presumed to be packaged because they persist over many passages (43).

Curiously, a SARS-CoV-2 genomic segment that contained almost all of the F3 sequence had not exhibited significant activity in the VLP assay system (25). Conversely, genomic fragments downstream of F3, including one encompassing PS9, did not support packaging when incorporated into the constructed DI RNA. One possible explanation for these divergent results could be that the VLP system actually measures nucleocapsid assembly, not gRNA packaging selectivity. The resolution of this apparent conflict must await construction of packaging-defective mutants in the viral genome, rather than in VLP or DI RNA surrogates.

### Other coronaviruses

Genomic packaging for the *Alphacoronavirus* porcine transmissible gastroenteritis virus (TGEV) was examined, as with the initial MHV PS studies, via characterization of packaged naturally arising DI RNAs (44). Construction of successively smaller DI RNAs ultimately showed that the TGEV PS is confined to the first 598 nt of gRNA (45, 46). This region was found to be refractory to further DI RNA deletion mapping, and it was concluded that the TGEV PS must consist of the entire 5′-most 598 nt of the genome, which would make it much larger and more complex than the MHV PS. Nevertheless, more subtle probing of this region through construction of point mutations in the virus would be desirable, especially in its three SL5 5′-UUCCGU-3′ loop motifs (22). Such an analysis may be challenging, however, because this same genomic segment also harbors 5′ *cis*-acting elements necessary for RNA replication (45).

For the *Gammacoronavirus* avian infectious bronchitis virus, the genomic requirements for packaging were analyzed through dissection of a naturally arising DI RNA. The resulting smallest packaged DI RNA contained a 1.1-kb segment of the 5′ end of the genome fused to a 1.2-kb segment of the nsp12 gene (47), but further localization of a potential PS element in either of these regions was not pursued. Finally, a set of five closely spaced stems displaying a repetitive loop motif has been noted at the nsp13–nsp14 junction in the gRNA of some *Deltacoronaviruses* (17, 48), but as yet, there is no experimental evidence that this structure serves as a PS.

## VIRAL COMPONENTS REQUIRED FOR RECOGNITION OF THE PS

The mechanism of selective gRNA packaging is not fully resolved, but there is general agreement that the viral N and M proteins are the essential participants. All work on this problem has been carried out with the *Embecovirus* MHV.

### Nucleocapsid protein

**Fig 4:**
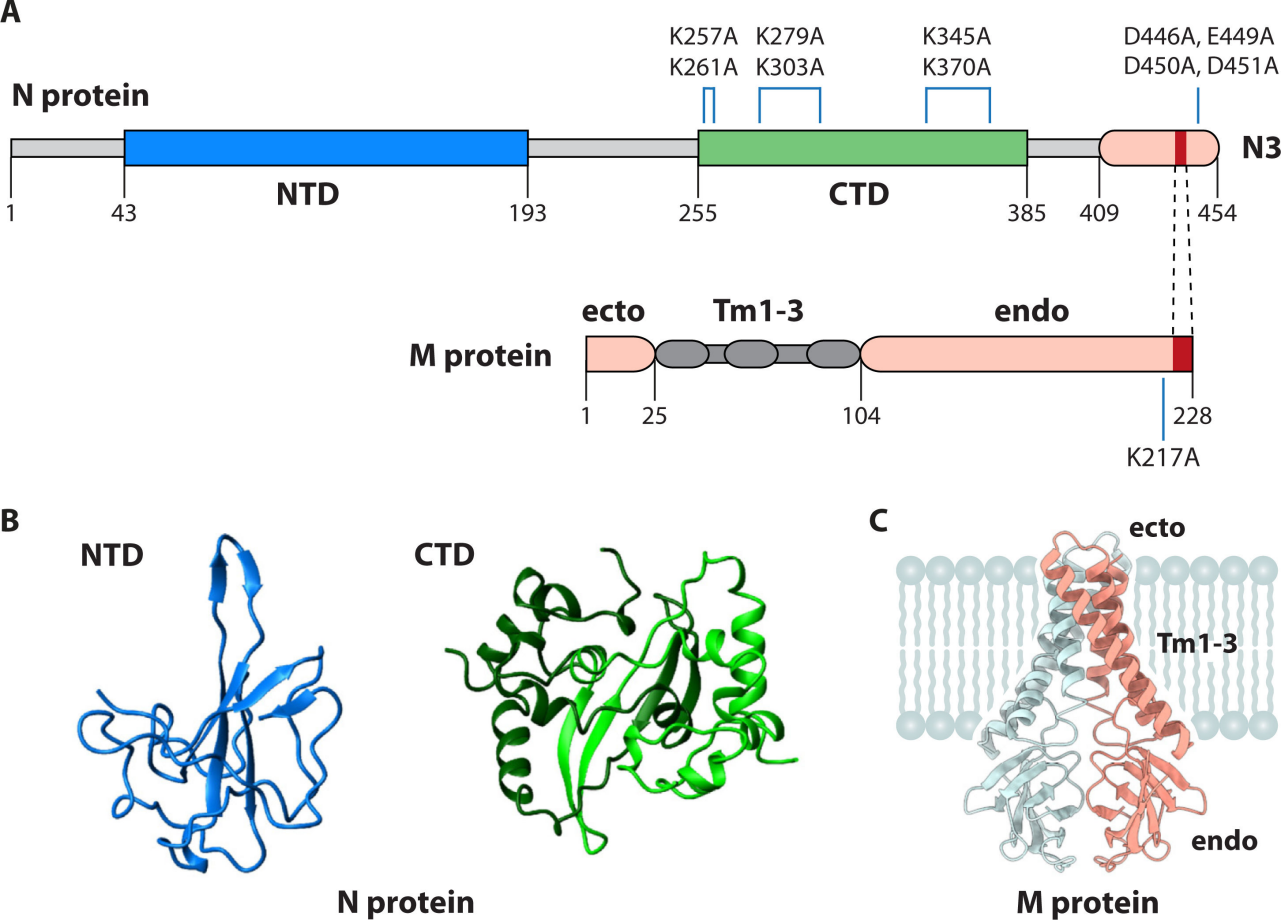
Components of PS recognition. (**A**) Linear diagrams of the N and M proteins of MHV. The N protein contains two structural domains that bind RNA (NTD and CTD) and a carboxy-terminal domain (N3). The CTD also mediates dimerization of the N molecule. At the amino terminus and connecting the three domains are three unstructured segments (in gray). Above the CTD, blue lines show the locations of three pairs of point mutations, each of which is sufficient to abolish selective gRNA packaging (55); the blue line above domain N3 marks the locus of a set of four point mutations that abolish selective gRNA packaging (55, 56). The M protein has a short amino-terminal ectodomain (ecto) linked to three transmembrane segments (Tm1–3) and followed by a long carboxy-terminal endodomain (endo). The blue line denotes the location of a point mutation that is sufficient to abolish selective gRNA packaging (55). In both N and M, regions that are critical for N-M virion assembly interactions are designated in red and connected by broken lines (50, 51, 57–59). Numbering indicates amino acid residues. (**B**) Crystal structures of the SARS-CoV-2 N protein NTD monomer and CTD dimer from Redzic et al. (60). (**C**) Cryo-EM structure of the SARS-CoV-2 M protein dimer from Dolan et al. (61). Images in panels **B** and **C** are reproduced under the terms of a Creative Commons Attribution 4.0 International License (http://creativecommons.org/licenses/by/4.0/).

The N protein wraps the viral genome into a beads-on-a-string ribonucleoprotein, and, as an RNA-binding protein, it is the most tangible candidate for a PS recognition determinant. Unusual among viral nucleocapsid proteins, the coronavirus N protein has two separate RNA-binding domains, designated the amino-terminal domain (NTD) and the carboxy-terminal domain (CTD) (Fig. 4A). The CTD also serves as a dimerization domain. At the actual carboxy terminus of the molecule is a third domain, N3, which is partially structured (49) and is the region of N that interacts with the M protein in virion assembly (50, 51). N3 also participates in one type of N-N interaction (49, 52). Flanking the NTD, CTD, and N3 are three largely disordered segments. Numerous NTD and CTD structures have been determined for *Alpha-*, *Beta-*, and *Gammacoronavirus* N proteins; all have the same architecture (Fig. 4B) despite the substantial divergence in their primary sequences. The NTD monomer presents a beta-platform with basic and aromatic amino acid residues postulated to form both electrostatic and stacking interactions with RNA. The CTD is a domain-swapped dimer with a rectangular slab shape displaying on one face a highly basic groove thought to interact with the phosphodiester backbone of RNA. Notably, a structure of the full-length N molecule, with or without bound RNA, is not yet available, although preliminary steps toward that goal have been reported for the N proteins of SARS-CoV-2 (53) and MHV (54).

In an initial exploration of the potential function of N protein in PS recognition, chimeric MHV mutants were constructed in which either the NTD or CTD of MHV was replaced with the corresponding domain from the SARS-CoV N protein (62). Since the SARS-CoV genome does not contain a counterpart of the *Embecovirus* PS in its nsp15 gene or elsewhere, it was expected that the SARS-CoV N protein would not have evolved the capability to recognize the MHV PS. Indeed, the CTD substitution mutant, despite containing a fully intact wild-type copy of the PS, was as defective in selective packaging as the silPS mutant. This outcome identified a principal role for the CTD in PS recognition. Contrary to this, the NTD substitution mutant maintained a completely wild-type packaging phenotype, indicating that the NTD is not directly involved in the selective packaging of gRNA. This result was somewhat surprising since, on the basis of structure, the NTD is often speculated to have a greater propensity for sequence-specific RNA binding. Subsequently, a finer mutational analysis targeted surface clusters of basic amino acids in the MHV CTD, based on their positions in the aligned SARS-CoV CTD structure (55). Some mutants created by this strategy were lethal or severely impaired, likely due to disruption of interactions required for assembly. However, three mutants were isolated that were entirely assembly-competent but exhibited a packaging-defective phenotype, incorporating large amounts of sgRNA into purified virions. Each of these contained two lysine-to-alanine mutations (Fig. 4A), thus delineating a set of positively charged residues that are necessary to allow the N protein to differentiate gRNA from other RNA molecules.

The results of the CTD genetic analysis were complemented *in vitro* through electrophoretic mobility shift assays (EMSAs) in which purified wild-type or mutant CTD constructs were bound to PS or silPS RNA substrates (55). It was observed that, in the presence of excess competitor tRNA, wild-type CTD preferentially formed a unique complex (complex 1) with the wild-type PS RNA substrate. This complex was absent from binding reactions with wild-type CTD and the silPS RNA substrate. Likewise, complex 1 was severely diminished or absent from binding reactions containing any of the three mutant CTDs and the wild-type PS RNA substrate. The formation of complex 1 was thus taken to represent sequence-specific RNA binding. Mirroring the genetic findings for selective packaging of gRNA into virions, formation of complex 1 in EMSAs was abolished either by mutation of the PS or else by mutation of particular residues in the CTD. To further discern the nature of specific RNA binding, the MHV CTD dimer structure was modeled using AlphaFold 3 (63). Like all other CTD structures, the MHV CTD possesses on one face a markedly basic groove of arginines and lysines that constitutes the site of nonspecific RNA binding (64–66). Notably, the pairs of lysine residues that were mutated in the three packaging-defective CTD mutants are situated on the edges of the CTD slab structure, lying peripheral to the basic groove. This suggested that sequence-specific RNA binding is determined by residues well separated from those that participate in general RNA binding. The main conclusion drawn from these genetic and *in vitro* analyses was that the CTD is the primary component of PS recognition (55).

Independent of the CTD, a second domain of the MHV N protein, N3, was also found to be crucial for the selective packaging of gRNA. In the otherwise highly basic N molecule, N3 stands out in having a preponderance of negatively charged residues. The packaging role of this domain was discovered through screening a collection of N3 mutants accumulated from other genetic studies (56). Among those mutants that were packaging-defective, the most localized was one in which four adjacent aspartate and glutamate residues were changed to alanines (Fig. 4A). These mutations mapped to a short interval downstream of two aspartates previously shown to be essential for assembly interactions with M protein (50). No subset of the four mutations was sufficient to abolish packaging competence, suggesting there is some functional redundancy in this critical cluster of negative charges (55). Significantly, the N3 mutant had an unaltered CTD and retained an intact wild-type PS. It also exhibited growth as robust as the wild-type virus, indicating that it was fully assembly-competent. This demonstrated that domain N3 has a function in the genomic packaging process separate from that of the CTD.

### Membrane protein

The M protein, the most abundant virion constituent (67), is a dimer with each monomer embedded in the membrane by three transmembrane domains. The major part of the molecule, the carboxy-terminal endodomain, is located in the virion interior (Fig. 4A). Recently determined structures for the M dimer depict the transmembrane region as a domain-swapped helical bundle connected to a pair of β-sandwich endodomain protomers (Fig. 4C) (61, 68, 69). The dimer endodomain can exist in either a short or a long conformation, the latter of which contacts the nucleocapsid and is associated with greater membrane curvature (70). The transition between the two forms is governed by a hinge region between the transmembrane and endodomain (68, 71). Most assembly interactions between the N and M proteins have been mapped to the extreme carboxy termini of each protein (Fig. 4A) (50, 51, 57–59), and these appear to correspond to the thread-like connections seen between N and M in ultrastructural studies of SARS-CoV and MHV virions (72, 73).

The essential function of M protein in PS recognition was first revealed in a series of pivotal experiments that carefully examined interactions between MHV M, N, and RNA in cells. Initially, a crucial M-N interaction was demonstrated by reciprocal coimmunoprecipitation analyses of infected cell lysates (74). It was shown through immunoprecipitation with anti-N antibody that N protein was bound to all viral RNA species, sgRNA as well as gRNA, confirming a previously perplexing observation that seemed incompatible with selective packaging (75, 76). However, this puzzle was resolved by the finding that anti-M antibody coimmunoprecipitated only the fraction of N that was bound to gRNA. Moreover, this M-N interaction could not be brought about simply by the co-expression of M and N in uninfected cells, indicating that some quality of gRNA was needed for it to occur. Further work established that the required gRNA element was the PS (13). Analysis of a collection of DI RNAs in infected cells, or else of nonviral RNAs in cells expressing N and M, showed that N protein bound to all RNA species, irrespective of the presence or absence of the PS. By contrast, only those RNA species containing the PS were immunoprecipitated by anti-M antibody and became packaged into virions. The remarkable culmination of this work was the discovery that M protein alone could interact with the PS in the absence of N protein (77). Nonviral RNA containing the PS (but not RNA lacking the PS) was incorporated into VLPs generated by the expression of the MHV M and E proteins. From this result, it was proposed that the M protein is the primary factor responsible for binding to the PS, and the interaction of M with the N protein in the nucleocapsid takes place subsequently.

Genetic evidence supporting an essential role for M protein in PS recognition has also recently been acquired (55). Although the coronavirus M protein is generally less tolerant to mutations than the N protein (71, 78), viable mutants were obtained, which targeted single basic amino acids in the M endodomain that are conserved among the *Embecoviruses* but not in other *Betacoronaviruses*. Some of these mutants grew robustly and were fully assembly-competent. One of them was found to be completely impaired in selective gRNA packaging despite having an intact PS—the same phenotype exhibited by N protein CTD and N3 mutants described above. This revealed that packaging could be abolished by a single point mutation in the M protein. This key residue (K217) is near, but separate from those to which M-N assembly interactions have been mapped (Fig. 4A).

Collectively, the studies discussed above point to four separate participants in the mechanism of specific gRNA packaging. These are the PS RNA element, situated in a region that is unique to gRNA and absent from sgRNAs (Fig. 2), and three protein components (Fig. 4A): the N protein CTD, the N protein domain N3, and the carboxy terminus of the M protein endodomain. The major unresolved issue is whether the N protein or the M protein plays the primary role in PS recognition. On the one hand, if the M protein is capable of binding to the PS independently of the N protein (77), then why do certain CTD mutations eliminate selective gRNA packaging while leaving assembly unaffected? On the other hand, if N protein binding to the PS initiates selective packaging (55), then what is unique about the single N-RNA complex containing the PS that allows it to outcompete other far more numerous N-RNA complexes for incorporation into budding virions? In a previous review (48), three models were set out in detail proposing PS recognition either (i) primarily by N or (ii) primarily by M, or (iii) mutually by a preformed complex of N and M. Current evidence does not yet allow an unequivocal choice among these alternatives. Future resolution of this issue may come from the development of more advanced assays for reconstitution of packaging *in vitro* and by structural determination of a complex of the PS with N and M.

## NUCLEOCAPSID ASSEMBLY

### Coronavirus nucleocapsid structure

**Fig 5:**
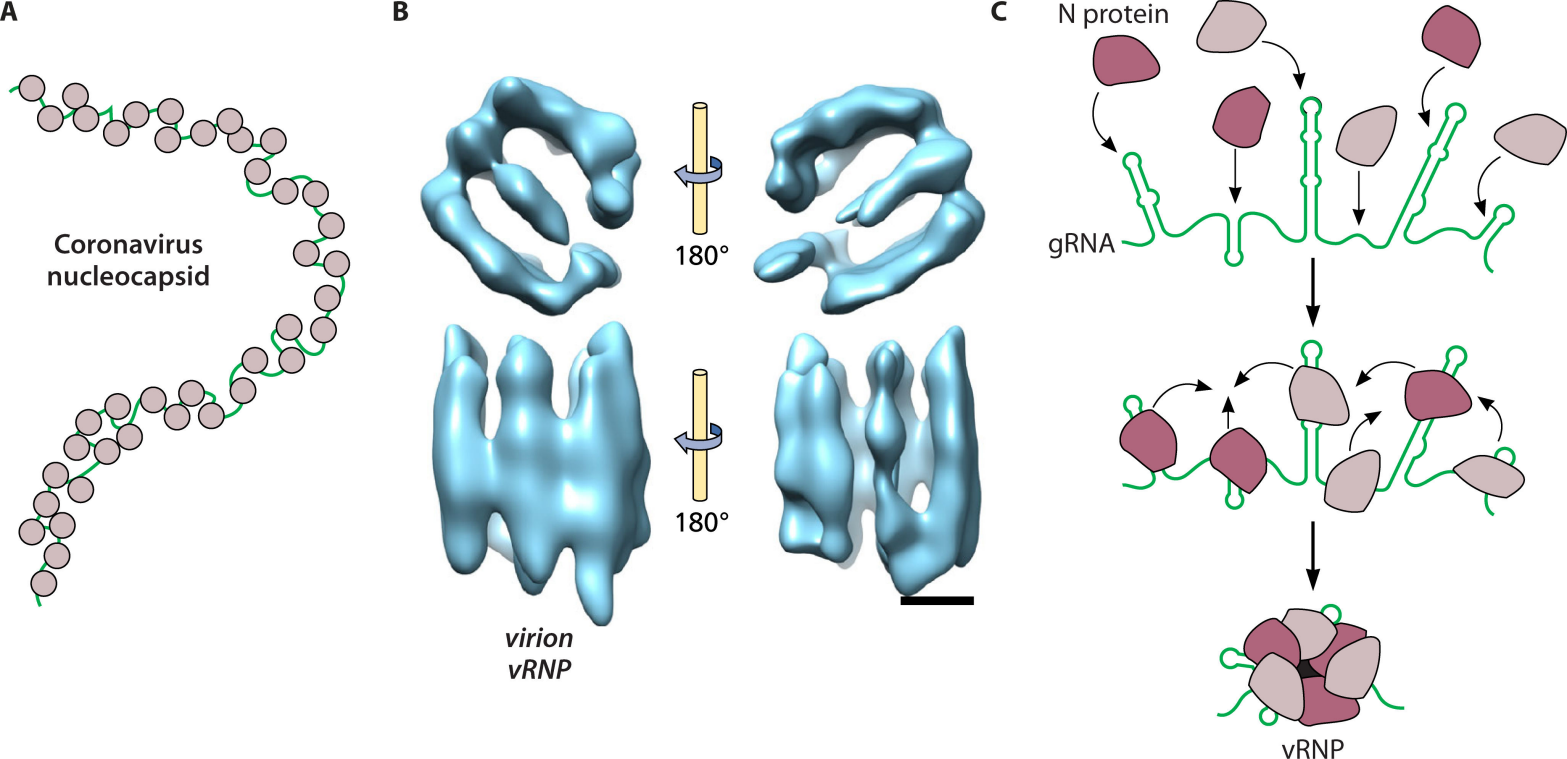
Coronavirus nucleocapsid structure and assembly. (**A**) Cartoon of a nucleocapsid released from a disrupted virion. (**B**) Top and side views of vRNP from SARS-CoV-2 virion visualized by cryo-ET; scale bar, 5 nm. Image from Klein et al. (80) reproduced under the terms of a Creative Commons Attribution 4.0 International License (http://creativecommons.org/licenses/by/4.0/). (**C**) Schematic of the proposed vRNP assembly mechanism in which individual dimers of N protein bind to gRNA and then coalesce into vRNPs via N–N interactions (84).

It was long believed, and recited in many papers and textbooks, that coronaviruses have helically symmetric nucleocapsids akin to those of nonsegmented negative-strand RNA viruses such as paramyxoviruses and rhabdoviruses. This notion came from early reports containing electron micrographic (EM) images of virions that had spontaneously disrupted or had been solubilized with nonionic detergents (Fig. 5A). However, a summary of that early work noted numerous gaps and points of disagreement that called for further critical analysis to elucidate the true morphology of the coronavirus nucleocapsid (reference 79 and references therein). Such critical analysis has very recently been provided by impressively detailed visualizations of SARS-CoV-2 virion structure by cryo-electron tomography (cryo-ET) (80, 81). In these studies, the nucleocapsid was modeled as a set of viral ribonucleoprotein (vRNP) complexes, connected by strands of RNA, arranged in a beads-on-a-string configuration. There are approximately 30–42 vRNPs per virion, with each vRNP estimated to contain 10–12 N protein monomers. These structures are heterogeneous, but low-resolution models of individual vRNPs obtained by subtomogram averaging depict them as cylindrical assemblies, having a quasi-circular base roughly 14–16 nm in diameter, with N molecules stacked to a comparable height as vertical pillars (Fig. 5B) (80, 81). Interestingly, structures similar to vRNPs were observed as circular densities in prior cryo-ET reconstructions of MHV virions, but these were interpreted as views of helical coils along the axis of the helix (73). In addition, with the benefit of hindsight, the best-resolved of the earliest EM images of nucleocapsids (82, 83) could now be taken to support the beads-on-a-string model.

### Nucleocapsid assembly interactions

The current basis for understanding coronavirus nucleocapsid assembly was established in a landmark study that reconstituted vRNP formation *in vitro* from purified SARS-CoV-2 N protein and synthesized fragments of gRNA (84). The complexes formed in this work were characterized by mass photometry and EMSA, and their stoichiometry was deduced to be 12 N monomers (6 dimers) bound to some 600 nt of RNA. Furthermore, negative-stained EM images of glycerol gradient-purified complexes revealed 15-nm structures that were highly similar in size and morphology to the vRNPs observed in the cryo-ET visualizations of intact virions. Less sturdy vRNPs of the same size and appearance were able to be formed between the N protein and a selected small (72 nt) genomic SL RNA, showing that a continuous stretch of 600 nt of gRNA was not required. This suggests that while RNA binding is necessary to form vRNPs, they are mainly stabilized by a multiplicity of N–N protein interactions (Fig. 5C). Examination of a set of N protein deletion mutants indicated critical roles in vRNP stabilization for domain N3 and the CTD, as well as for the linker region between the NTD and CTD. This result accords well with the many interdomain N–N interactions that had previously been mapped in studies using genetics, immunoprecipitation, or cross-linking mass spectrometry (62, 85, 86). Like their counterparts in virions, *in vitro*-assembled vRNPs were seen to be heterogeneous. Since much of the higher-order structure of coronavirus gRNA is retained within virion particles (29, 87), this heterogeneity of vRNPs was postulated to reflect the diversity of RNA secondary structures that each contains (84).

Various details of the assembly process remain unresolved. Based on the linear morphology of nucleocapsids released from virions (82, 83), vRNPs most likely form successively along gRNA, perhaps as the gRNA transits from the RNA synthesis compartment to the cytoplasm (see below). However, an alternative possibility is that vRNPs are hubs uniting discontiguous regions across the genome (84). Also unknown at present are the lengths of unbound RNA strands between successive vRNPs, and whether there is a significant pool of N protein in virions that is not directly associated with RNA. What is clear, nonetheless, is that nucleocapsid assembly initiates at multiple sites on gRNA. Although N exhibits some preference for structured gRNA, vRNP formation does not appear to require specific RNA sequences or structural elements. This finding further reinforces the conclusion that coronavirus nucleocapsid assembly is a separate process from selective gRNA packaging.

### N phosphorylation and nucleocapsid assembly

**Fig 6:**
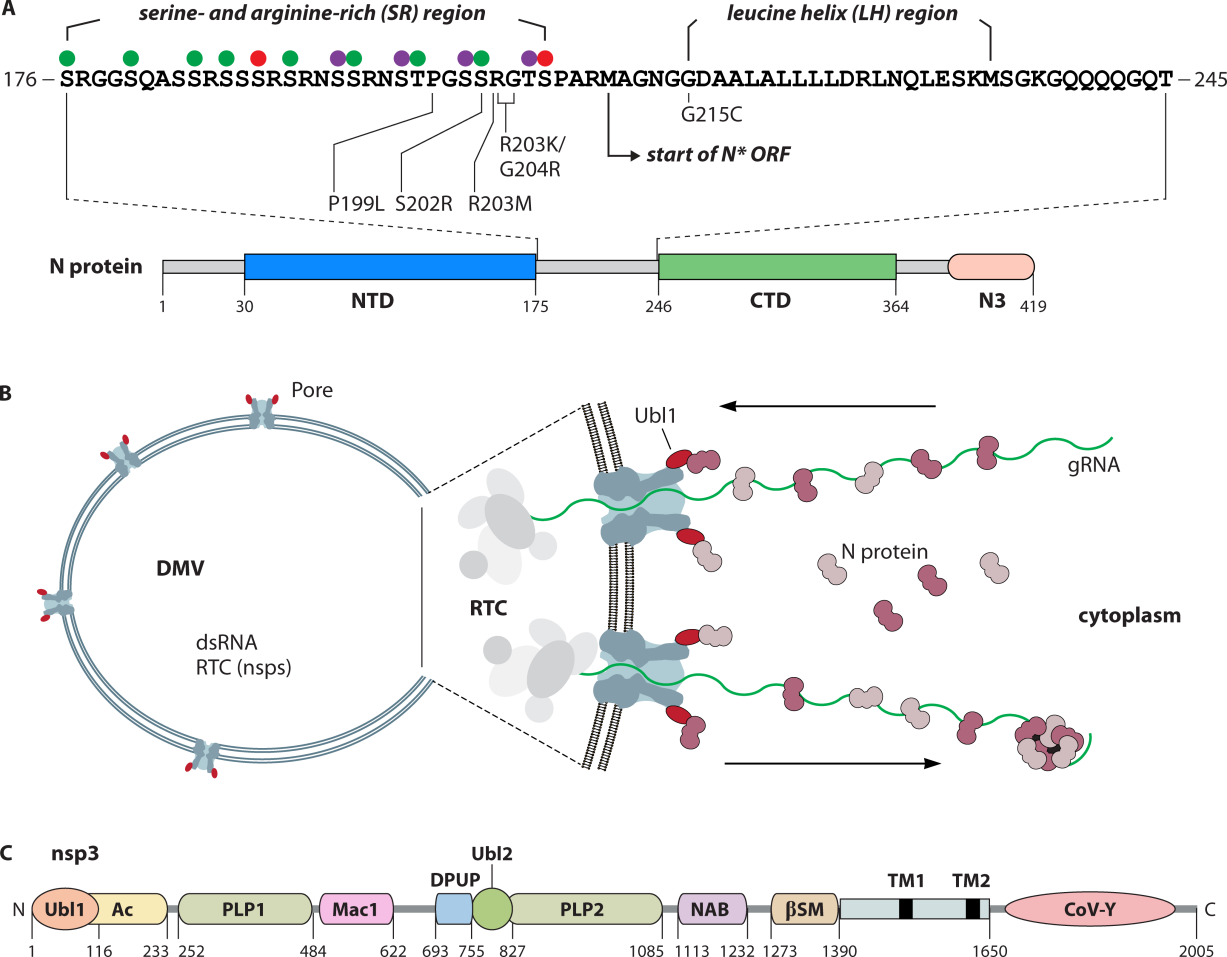
N protein phosphorylation and interaction with nsp3 at the double-membrane vesicle (DMV) pore. (**A**) Linear diagram of the N protein of SARS-CoV-2; numbering indicates amino acid residues. The expanded segment shows the sequence of the central linker between the NTD and CTD containing the SR region and the leucine helix (LH) region. Closed circles above the SR region indicate residues that become phosphorylated sequentially by the protein kinases SRPK1/2 (red), GSK-3 (green), and CK1 (purple) (92). Shown beneath the sequence are mutations from evolved SARS-CoV-2 variants that affect N protein function. Also indicated is the start of the open reading frame for the amino-terminally truncated N* protein that results from a novel sgRNA in viruses containing the R203K/G204R dual mutation (94, 95). (**B**) Model of the DMV that contains the coronavirus RTC, as visualized for MHV-infected cells (96). Spanning both membranes are multiple copies of the pore, composed predominantly of nsp3, which serves as a channel between the DMV interior and the cytoplasm. The amino-terminal Ubl1 domain of nsp3 is shown in red. In the expansion to the right are shown schematics of how the pore might function to deliver gRNA into the DMV at the outset of infection (top) and later in infection to export newly synthesized gRNA out of the DMV to assemble with N protein into progeny nucleocapsids in the cytoplasm (bottom). (**C**) Linear diagram of MHV nsp3; numbering indicates amino acid residues. The domains of nsp3, discussed in detail elsewhere (97, 98), are: Ubl1 and 2, ubiquitin-like domains; Ac, hypervariable acidic region; PLP1 and 2, papain-like proteases; Mac1, macrodomain 1; DPUP, domain preceding Ubl2 and PLP2; NAB, nucleic acid binding domain; βSM, *Betacoronavirus*-specific marker; TM1 and 2, transmembrane segments; CoV-Y, coronavirus highly conserved domain. Portions of the modular structure of nsp3 can vary; of note, *Sarbecoviruses* lack PLP1 but have two additional macrodomains (Mac2 and Mac3) following Mac1.

The N protein rapidly becomes phosphorylated to varying degrees following its synthesis (88, 89), but until recently, the possible function of phosphorylation was largely speculative. Studies performed in the wake of the emergence of SARS-CoV-2 have provided a bounty of new details about how N acquires phosphates and what may be the role of this post-translational modification. Although there are multiple sites in the N protein that can be phosphorylated, the foremost concentration of phosphoserines and phosphothreonines is found in the serine- and arginine-rich (SR) region of the central linker between the NTD and the CTD (Fig. 6A) (90–92). The SR region is frequently said to be highly conserved among coronavirus N proteins, but this is correct only in the sense that all coronavirus N proteins contain an SR region. The primary sequences of SR regions can be quite variable, even between closely related viral species (93), and the evolution of this region in SARS-CoV-2 variants has had significant consequences. For SARS-CoV-2, exhaustive mapping of kinase substrate specificity led to the identification of the relevant protein kinases and deconvolution of the phosphorylation pathway of the N protein SR region (92). It was shown that phosphorylation of this region proceeds through a cascade of three protein kinases—SR-rich splicing factor protein kinase 1 or 2 (SRPK1/2), glycogen synthase kinase-3 (GSK-3), and casein kinase I (CK1)—acting in that obligate order. Because the substrate recognition motifs of GSK-3 and CK1 each include a phosphoserine or phosphothreonine residue, their actions depend upon priming by the prior protein kinase in the cascade (SRPK1/2 or GSK-3, respectively). Therefore, the most highly phosphorylated form of the SARS-CoV-2 N SR region can contain as many as 14 phosphates within a span of only 31 amino acids (92) (Fig. 6A).

From earlier work with BCoV and SARS-CoV, it was known that the intracellular N protein is *hyper*phosphorylated, and conversely, the N protein contained in virions is *hypo*phosphorylated (99, 100). This functional divergence was replicated by the properties of *in vitro* assembly of SARS-CoV-2 vRNPs (84). If assembly reactions, normally performed with (bacterially expressed) unphosphorylated N, were instead carried out with N protein that had been phosphorylated *in vitro*, vRNP formation was inhibited. Moreover, vRNPs formed with a phosphomimetic N mutant, containing aspartates in place of 10 phosphorylation targets in the SR region, had an extended heterogeneous structure differing from the compactness of wild-type vRNPs. A number of other studies have broadened the scope of this result through examination of SR region mutations that arose in circulating SARS-CoV-2 variants. N proteins containing each of four such single mutations—P199L, S202R, R203M, and R203K—enhanced assembly 10-fold in the VLP system described above (25, 94). The same N mutants exhibited reduced phosphorylation when expressed in a human cell line. Thus, the extent of N protein phosphorylation inversely correlated with VLP assembly: mutants with reduced phosphorylation supported increased assembly. In opposition to this, wild-type N protein and other N mutants constructed as controls had comparatively reduced amounts of assembly, which were boosted to high levels in the presence of a GSK-3 inhibitor. Additionally, reverse-genetically engineered viruses containing either the S202R or the R203M mutation grew to 50-fold higher titers than the wild type (i.e., the ancestral WA1 SARS-CoV-2 isolate) and had increased fitness, as measured by viral competition assays in human cells (25).

Of particular interest has been the SR region dual mutation R203K/G204R (Fig. 6A). This co-occurring pair of changes became fixed in a SARS-CoV-2 lineage that was a predecessor to the alpha, gamma, and omicron variants. Constructed R203K/G204R mutant viruses were found to outcompete the wild-type virus in human cells and had heightened replication in hamsters, producing greater disease severity (101, 102). In addition, in co-infection competition experiments, R203K/G204R mutant viruses showed increased fitness compared to the wild type. N proteins containing the R203K/G204R mutation gave enhanced yields of VLPs and had reduced phosphorylation in cells, thereby further confirming the reciprocal relationship between phosphorylation and assembly (94). But the R203K/G204R mutation was found to have an unexpected second property: the same mutations that alter the N coding sequence create a new transcription-regulating sequence midway through the N gene (103). This results in the synthesis of an additional sgRNA encoding a truncated version of the N protein, designated N* or N.iORF3 (95). N* lacks the amino-terminal 129 amino acids of N, including the NTD and the entire SR region (Fig. 6A). Remarkably, it was demonstrated that this shortened form of N protein was sufficient for vRNP formation (104). The addition of purified N* protein to a gRNA fragment led to the assembly of vRNPs that, by EM, were very similar to those formed with full-length N. This revealed that the NTD and SR region are dispensable for assembly, leaving the CTD as the sole essential RNA-binding domain in vRNPs. Consistent with the effect of phosphorylation on assembly, vRNP formation was inhibited by appending a phosphomimetic SR region to the N* protein. Moreover, N*, which cannot be phosphorylated, was able to support VLP assembly much better than full-length N (94). Since the N* protein is expressed at less than 2% of the level of full-length N (95), it is unclear whether it significantly modifies virion assembly, but nevertheless, N* has revealed some important characteristics of the N protein. Overall, the lessons learned from SR region mutants are as follows. First, even a small number of mutational changes can rearrange successive priming-dependent protein kinase recognition motifs and consequently disrupt the full phosphorylation cascade. Second, changes in the SR region alter the balance between hypophosphorylation, which promotes virion assembly, and hyperphosphorylation, which promotes some other function of N related to RNA synthesis. It has been proposed that SARS-CoV-2, in the course of the COVID-19 pandemic, evolved to fine-tune this balance for optimal viral replication and transmission (94).

A second segment of the N protein central linker, the leucine helix (LH) region (Fig. 6A), also contributes to nucleocapsid assembly, but its role is less well defined. The LH region, a stretch of 20 amino acids downstream of the SR region, was identified as one of the segments of N critical for *in vitro* formation of vRNPs (84). Evidence from biophysical studies of the SARS-CoV-2 LH peptide suggested that, under certain conditions, it could form a self-associating alpha helix that was proposed to stabilize an N–N interface (105). Relative to this, considerable attention has been devoted to an N protein mutation, G215C, which became fixed in later lineages of the SARS-CoV-2 delta variant. This mutation, which falls at the upstream edge of the LH region (Fig. 6A), has been found to promote N protein oligomerization *in vitro* (104, 106) and enhance viral titers in an engineered SARS-CoV-2 recombinant (107). However, reports differ as to whether these effects result from the formation of a disulfide bond within or between N dimers, or else from a purely noncovalent interaction. Intriguingly, G215C mutant virions were found to contain higher amounts of N protein than the wild type, and some of them appeared to have an elongated morphology (107).

### Compartmental separation of RNA synthesis and nucleocapsid assembly

In contrast to the more straightforward connection between hypophosphorylation of N protein and nucleocapsid assembly, the current understanding of the association between N hyperphosphorylation and RNA synthesis is somewhat nebulous. Many RNA synthesis-assisting activities previously ascribed to N protein were based on *in vitro* assays or on more general effects *in vivo* that could not clearly be attributed to either gRNA replication or sgRNA transcription. Moreover, some of these putative activities appear to be ruled out by what is now known about the compartmentalization of viral proteins during infection.

The machinery of coronavirus RNA synthesis, the RTC, comprises most of the 16 nsps that are processed from the huge polyproteins pp1a and pp1ab at the outset of infection (1, 108). The RTC includes an RNA-dependent RNA polymerase (nsp12) and associated processivity factors (nsp7 and nsp8); a helicase (nsp13); 5′ capping enzymes and cap methyltransferases (nsp12, nsp9, nsp14, and nsp16); a proofreading 3′–5′ exonuclease (nsp14); proteases (nsp5 and domains of nsp3); and an endonuclease (nsp15). This collection of RNA-synthetic enzymatic activities becomes sequestered within a network of interconnected membranous structures, which are induced during the earliest stages of coronavirus infection by the three membrane-bound RTC subunits nsp3, nsp4, and nsp6 (97, 109, 110). The resulting dramatic intracellular rearrangements create a variety of membrane structures: double-membrane vesicles (DMVs), double-membrane spherules, and convoluted membranes. Among these, DMVs have been shown to be the only structures that contain double-stranded RNA (dsRNA), an intermediate of viral RNA synthesis (109–111). More definitively, it was demonstrated by EM autoradiography of RNA labeled with tritiated uridine that DMVs are the sites of active viral RNA synthesis; little or no RNA synthesis could be detected in other membrane structures (111). Critically, no ribosomes are found within DMVs, nor do they contain N protein (96, 112). RNA filaments in the DMV interior imaged by cryo-ET appear smooth and have a diameter consistent with an A-form dsRNA helix (80). They are too narrow to be decorated with N protein and are wholly different from the vRNP structures inside virions.

The entirely closed nature of DMVs made it challenging to account for how RNA and nucleotide precursors could enter and exit these compartments. This apparent incongruity was settled by the stunning discovery that each DMV contains multiple copies of a molecular pore spanning both membrane bilayers and connecting the interior space to the cytoplasm (96) (Fig. 6B). The pore structure, resolved by high-resolution cryo-ET of MHV-infected cells, was visualized as a hexameric crown-like assembly standing atop a membrane-embedded platform. The major molecular constituent of the pore was shown to be nsp3, a multi-domain protein that is by far the largest RTC component (97, 98, 113) (Fig. 6C). Almost all of nsp3 (except the two transmembrane domains) resides on the exterior of the DMV. Each of six nsp3 monomers projects into the cytoplasm, with its amino-terminal ubiquitin-like domain 1 (Ubl1) as one of the points of the crown. Strikingly, clusters of vRNPs were observed in cytoplasmic regions adjacent to these external DMV projections (96). The channel running through the pore has a diameter sufficient to accommodate single-stranded RNA but is too narrow for the passage of vRNPs or even N protein dimers. The architecture of this complex was confirmed (114) and further elaborated (115) for DMVs formed by the co-expression of only nsp3 and nsp4 of SARS-CoV-2. Notably, the model obtained in the latter study showed that the pore is lined with three levels of rings of positively charged residues of nsp3 and nsp4, which were suggested to participate in RNA transit. Mutations of some of these amino acids did not disrupt pore integrity but were lethal in constructed viral recombinants.

The separation of the RTC, within DMVs, from the N protein, in the cytoplasm, has important implications for how the N protein might contribute to viral RNA synthesis. First, previous lower-resolution observations of the co-localization of a fraction of N protein with subunits of the RTC (116–119) were likely due to N interacting with nsp3 at the external face of DMVs. Second, the helix-destabilizing and strand-annealing activities of N protein (120–122) studied *in vitro* cannot promote the template strand-switching that brings about fusion of the leader and body RNA segments during negative-sense sgRNA transcription. However, these RNA chaperone properties of N may very well have important functions in the cytoplasm. Third, putative *in vitro* interactions between N and the nsp12 RNA-dependent RNA polymerase (123, 124) have no clear biological relevance, since nsp12 and N protein do not encounter each other during infection.

Any direct interaction of the N protein with the RTC is restricted to its contact with nsp3. The interaction between N and nsp3 was discovered through an attempt to replace the MHV N protein SR region with the closely related SR region from BCoV (93). Viral recombinants constructed with this substitution were extremely impaired but were rescued by second-site mutations in either the N SR region or in the amino-terminal domains of nsp3: Ubl1 and the adjacent hypervariable acidic region (Ac) (Fig. 6C). Similarly, a mutant substituting the SARS-CoV SR region into the MHV N protein could be recovered only if it had second-site reverting mutations in either the SR region or the MHV Ubl1 domain. In additional support of this interaction, it was shown that a glutathione S-transferase (GST)-Ubl1-Ac fusion protein pulled down N protein from an MHV-infected cell lysate (93). To more broadly explore whether other parts of the RNA-synthetic machinery interact with N protein, an all-inclusive yeast two-hybrid (Y2H) analysis was performed between the MHV N protein and each of the RTC subunits nsp1–nsp16 (125). This established that N interacts only with nsp3, and in particular, with the Ubl1-Ac domains of nsp3.

Further studies have mapped the N-nsp3 interaction by a variety of methods, but these have not yielded fully concordant conclusions. Most genetic results point to Ubl1 alone as the crucial nsp3 participant. An MHV mutant containing a substitution of the BCoV N protein was completely complemented by a constructed nsp3 chimera containing the BCoV Ubl1 and Ac domains, as well as by a chimera containing just BCoV Ubl1 (126). Consistent with this, numerous MHV point and deletion mutations in Ubl1 were lethal, whereas the Ac domain could be entirely deleted with no effect on viral phenotype (126, 127). On the other hand, an assay of the colocalization of SARS-CoV-2 N and nsp3 constructs expressed in human cells suggested that optimal interaction with N requires both the Ubl1 and Ac domains of nsp3 (128). For the N protein, although the initial genetic work pointed to the SR region, subsequent reports indicated that other parts of the N molecule are also involved. Both Y2H and GST pulldown assays mapped separate interactions with Ubl1-Ac to the N protein NTD and to the central linker (which encompasses the SR and LH regions) (125). A study that used isothermal calorimetry (ITC) to measure the N-nsp3 interaction observed high-affinity binding of MHV Ubl1 to an NTD-SR construct, with the major contribution coming from the SR region (129). Essentially, the same results were obtained by ITC with SARS-CoV-2 Ubl1 binding to a set of N deletion mutants (128). Optimal binding was found with a construct of the NTD plus the central linker, but there was also a 20-fold lower binding to the NTD alone; all other segments of N could be deleted without consequence. A crystal structure of a complex of the SARS-CoV-2 NTD and Ubl1 modeled the NTD wedged between two monomers of Ubl1 in a V-like configuration, with the Ubl1 monomers connected to each other by short amino-terminal beta-strands (128). One crystal structure of SARS-CoV-2 Ubl1 alone also showed it as a V-shaped dimer (130). However, this is hard to reconcile with the geometry of the DMV pore, in which the six diverging points of the hexameric crown structure each terminate in a monomeric Ubl1 domain (96). By contrast, in other crystal and NMR structures of Ubl1—from SARS-CoV, MHV, and SARS-CoV-2—this molecule is monomeric, and its amino-terminal segment is flexibly disordered (129, 131, 132).

Multiple studies have charted the interplay between N phosphorylation, the N-nsp3 interaction, and RNA binding. An inverse relationship between RNA binding and binding to Ubl1 was implied by the enhancement by RNase treatment of GST-Ubl1 pulldown of N protein from lysates of cells infected with wild-type MHV or certain SR region mutants that were constructed to ablate or mimic phosphorylation (93, 127). Similarly, in the ITC analysis of the MHV N-nsp3 interaction, the formation of a high-affinity Ubl1-NTD-SR complex was inhibited by prior binding of the NTD to RNA, suggesting that Ubl1 and RNA compete for binding to the NTD (129). Likewise, SARS-CoV-2 Ubl1 was shown by EMSA to compete with a short RNA substrate for binding to N protein (128). At least in part, these findings may be explained by the results of a study that followed the dynamics of SARS-CoV-2 N protein phosphorylation by NMR. This work found that binding of short RNAs by a bacterially expressed (hypophosphorylated) construct of the NTD and central linker was totally abolished by *in vitro* hyperphosphorylation of the SR region by the relevant protein kinases (SPRK1, GSK-3, and CK1) (133). Moreover, evidence was presented that this inhibition of RNA binding was brought about by direct interaction of the hyperphosphorylated SR region with the RNA-binding surface of the NTD.

There clearly remain abundant details to be settled, but the wealth of new information from the studies discussed above appears to be converging on a model of phosphorylation providing a temporal switch modulating both the binding of N protein to RNA and to Ubl1 at the exterior (cytoplasmic) face of the DMV pore (Fig. 6B). At the earliest stages of infection, the N-Ubl1 interaction might serve to localize gRNA at the RNA synthesis compartment. Hyperphosphorylation would enable the removal of N from gRNA and assist in the insertion of the genome into the DMV to serve as the template for transcription and replication. One possibility is that multiple cycles of N protein phosphorylation and dephosphorylation act processively to ratchet gRNA through the pore into the DMV. Conversely, later in infection, a pool of hypophosphorylated N in the vicinity of Ubl1 would serve to assemble vRNPs as newly synthesized gRNA emerges from the DMV. Energetically expensive ratcheting might not be required at this stage. Ultrastructural studies show interior sections of the pore making contact with other RTC components, suggesting that RNA synthesis could drive export of gRNA and sgRNA through the pore to the cytoplasm (96, 134). Considerable additional work will be needed to build on this or other speculative scenarios.

## FUTURE RESEARCH CHALLENGES

A number of areas of coronavirus genome packaging and nucleocapsid assembly, some of them listed here, are well suited for future exploration. One of the foremost would be to precisely define PSs beyond those of the *Embecoviruses*, especially for the highly pathogenic human coronaviruses. This can be accomplished most definitively through viral mutant construction and virion purification. For the SARS-CoV-2 PS candidates F3 (8) and PS9 (25), each of which falls in the rep1b open reading frame (Fig. 3C and D), it might be required to apply various recoding strategies within very narrow intervals. Such work, despite being labor-intensive and requiring higher biosafety containment, has the potential to produce both important new insights and novel antiviral molecular targets. Linked to the problem of PS identity is elucidation of the detailed mechanism of recognition of the PS by virion protein components. It is clear that vRNP assembly occurs independently of the PS. What is special about the particular vRNP that contains the PS, and how does its presence lead to the exclusion of sgRNAs and cellular RNAs from budding virions? The kinetics of the interplay among the CTD and N3 domains of the N protein and the endodomain of the M protein, as well as the compositions of the complexes involved, remain open questions.

A major unresolved question is whether the set of interacting N protein molecules in vRNPs has a defined architecture. One interpretation of the observed heterogeneity of vRNPs is that it is due to the wide range of gRNA structures they contain (84). If this is correct, then it might be possible to find conditions and artificial RNA substrates or analogs that promote the assembly of more uniform particles suitable for higher-resolution cryo-EM analysis. Alternatively, a more variable arrangement might be indicated by the finding that vRNPs can sometimes accommodate more than six N protein dimers and that they can form in the absence of the NTD (84, 104). If so, then what does this say about the plasticity of N–N interactions in vRNP assembly and whether this poses a barrier to their disruption by antiviral drugs? Related to this, meaningful employment needs to be found for the NTD of the N protein. The NTD structure is highly conserved among all coronaviruses (64), and mutations that negate its RNA-binding ability are lethal to the virus (121, 135). However, it is dispensable for vRNP assembly (94, 104), and it is not a determinant of PS recognition (62). It has been noted that, while the CTD structure is held in common, there is no counterpart of the NTD in the N proteins of viruses in other families in the *Nidovirus* order, including many that carry out leader-body fusion to produce sgRNAs (113). Thus, it remains to be discovered why the coronavirus NTD is essential. A further basic issue in N protein biochemistry is that it is unlikely that phosphorylation is a switch that can only be turned on but not off. A significant gap to address in appreciating the role of this modification is the need to identify and characterize the regulation of cellular protein phosphatases that dephosphorylate SR region residues. These would be expected to counterbalance the action of protein kinases in the modulation of N protein function. In this respect, it is intriguing that there is a consensus protein phosphatase 1 docking motif, (K/R)(K/R)(V/I)X(F/W) (136), conserved at the flexibly disordered amino terminus of the nsp3 Ubl1 domain in most *Betacoronaviruses*.

More broadly, a very desirable goal would be the delineation of the pathogen-associated molecular patterns displayed by viruses that fail to carry out selective gRNA packaging and how these patterns are sensed (21). In addition, the particular downstream pathways that become activated are as yet undefined, nor is it clear whether these point to interferon-stimulated activities that are not already known to be inhibited by the panoply of coronavirus proteins that antagonize host cell innate immunity (137). Finally, a deeper understanding of both packaging and assembly will have the potential to guide the design and selection of therapeutics for highly pathogenic human coronaviruses. Well-defined N–N or N–M interfaces that are repeated in molecular assemblies would be opportune candidates for dominant drug targets that can suppress drug-resistant variants (138). PS structures, themselves, may become targets for small-molecule antiviral compounds as the field of RNA therapeutics continues to advance (139, 140).
